# Biological Maturation and Hormonal Markers, Relationship to Neuromotor Performance in Female Children

**DOI:** 10.3390/ijerph17093277

**Published:** 2020-05-08

**Authors:** Paulo Francisco de Almeida-Neto, Paulo Moreira Silva Dantas, Vanessa Carla Monteiro Pinto, Tatianny de Macêdo Cesário, Nathália Monastirski Ribeiro Campos, Eduardo Estevan Santana, Dihogo Gama de Matos, Felipe J Aidar, Breno Guilherme de Araújo Tinoco Cabral

**Affiliations:** 1 Department of Physical Education—Federal University of Rio Grande do Norte, Natal UFRN59064-741, RN, Brazil; pgdantas@icloud.com (P.M.S.D.); vanecmpinto@gmail.com (V.C.M.P.); tatiannymc@gmail.com (T.d.M.C.); nathaliacampos07@hotmail.com (N.M.R.C.); edusantana.93@gmail.com (E.E.S.); brenotcabral@gmail.com (B.G.d.A.T.C.); 2 Institute of Parasitology, Mcgill University, Montreal, QC H3A 0G4, Canada; dihogogmc@hotmail.com; 3 Group of Studies and Research of Performance, Sport, Healt and Paralympic Sports—GEPEPS, The Federal University of Sergipe, UFS, São Cristovão 49100-000, Sergipe, Brazil; fjaidar@gmail.com; 4 Department of Physical Education, Federal University of Sergipe—UFS, 49100-000 São Cristovão, Sergipe, Brazil; 5 Graduate Program in Master’s Level at Department of Physical Education, Federal University of Sergipe—UFS, São Cristovão 49100-000, Sergipe, Brazil; 6 Graduate Program in Master and Doctorate’s Level at Departament of Physiological Science, Federal University of Sergipe—UFS, São Cristovão 49100-000, Sergipe, Brazil

**Keywords:** estradiol, testosterone, sports performance, young

## Abstract

*Background*: Mechanisms that influence muscle strength can interfere with neuromotor performance and overall health, thus hormone markers and maturation can interact in this process. Objective: The present study aimed to verify the relationship of hormonal markers and biological maturation on neuromotor abilities in young people. *Methods*: This is a cross-sectional study with 44 female participants (11.5 ± 1.5 years). Hormones were analyzed biochemically. Skeletal and somatic maturation were analyzed using anthropometry. The muscular power of the upper and lower limbs, body speed with change of direction, and speed of the upper limbs were verified. *Results*: Bone age was correlated with hormonal markers (estradiol: r = 0.58; *p* = 0.0007), (testosterone: r = 0.51; *p* = 0.005). Peak growth velocity correlated with estradiol (r = 0.51; *p* = 0.004). The power of the lower limbs (estradiol: r = 0.52; *p* = 0.006; testosterone: r = 0.42; *p* = 0.03) and of the upper limbs (estradiol: r = 0.51; *p* = 0.007; testosterone: r = 0.42; *p* = 0.02) had a positive correlation with hormone levels and had similar results with maturation. The analysis by artificial neural networks indicated that the maturation can predict the neuromotor performance between 57.4% and 76%, while the hormonal markers showed a potential of more than 95% for the foreshadowing of the neuromotor performance of the upper limbs. *Conclusion*: It was possible to conclude that the hormones had a relationship with maturational development and bone age in female subjects.

## 1. Introduction

Biological maturation is a natural lifelong process that promotes morphophysiological changes in individuals [1]. Biological age is, therefore, defined by the individual maturation rhythm of each person. Thus, biological maturation depends on genetic, nutritional, and environmental factors; thus, the improvement of neurological, endocrine, bone and muscular systems that occur throughout life [1,2]. During this process, puberty is a milestone due to the occurrence of faster maturity, mediated by the increase of estrogen group steroid hormones [3,4,5,6]. This group of hormones play an important role in female biological characteristics and may influence the onset of menstrual period and the development of secondary sexual characteristics [7,8,9].

Typically, growth hormones reach a peak during the pubertal process [1,2,10,11]. In females, the growth hormone peak activates the hypothalamic–pituitary–ovary axis, promoting the production of estrogens (estradiol, estrone, estriol, and androstenedione) [1,4,12,13]. It is worth mentioning that among the estrogens produced by the female organism, this organism stands out for having an intense link with the reproductive function, and it is during the puberty phase that, among the functions in the organism, its production is accentuated. This is in addition to providing stimulation of the release of eggs by the ovarian follicles and acting on the fallopian tubes (i.e., female organ) by stimulating muscle contractions in the endometrium, raising its levels in the female menstrual phase [3,4,5,7].

Previous studies have demonstrated a relationship of maturation with neuromotor performance, exposing that biological maturation can influence the vertical jump skills, countermovement jump, strength of the upper limbs, in the neuromotor control of the muscles involved in the trunk region [14,15,16,17]. Furthermore, within sports, maturation can influence the quality of execution of specific skills (i.e., performance in Olympic rowing, basketball and volleyball, among others) [14,15,16,17,18,19]. Besides that, an increase in estradiol concentrations throughout the maturation process and the accurate development of the neuromotor system. In other words, the interaction between nerve impulses and the skeletal muscles that generate movement increases, leading to maximal voluntary strength of the biceps brachii and knee extension [20,21].

In addition, a linear association between sex hormone levels and strength development has been suggested. The association can be explained by the interaction of sex hormones with protein synthesis, which results in an increase in the lean mass and contributes to gain in muscle strength [22,23,24]. Additionally, due to the abundance of hormones at puberty, especially in female participants [4], intramuscular hormone receptors for estrogen hormones such as estradiol, for example, frequently act in the regulation of metabolism [25].

Moreover, the metabolism ends up being stimulated by several mechanisms such as muscle contraction generated by neuromotor activities that require more recruitment of type II muscle fibers (i.e., fast-twitch muscle fibers stimulated by the anaerobic energy pathway; and muscle fibers stimulated predominantly in muscle power activities). In this context of biological systems altered by maturation, the skeletal muscle process tends to gain prominence due to constant changes due to the human growth process [26].

In this sense, it is likely that one of the mechanisms related to strength gain during the maturational process is hormone-mediated [25,26,27]. Thus, in view of the foregoing, our hypothesis is that the maturational stages and levels of hormone markers in the body may be significantly related to the performance of neuromotor skills. Therefore, the objective of the present study was to verify the relationship between hormonal markers and biological maturation markers on the neuromotor capacities of young females.

## 2. Materials and Methods

### 2.1. Subjects

The study was descriptive, with a cross-sectional design. The sample consisted of 44 girls (11.5 ± 1.5 years). In this study, participants were chosen in a non-probabilistic and intentional manner. The participants were part of a social sport initiation project in the city of Natal, Rio Grande do Norte, Brazil. As inclusion criteria, the volunteers were between 10 and 12 years of age, were female, and did not have clinically diagnosed hormonal dysfunctions. All who were taking food or hormonal supplementation or who had performed vigorous activity 24 h before the tests were excluded.

The research was analyzed and approved by the Research Ethics Committee—of the Federal University of Rio Grande do Norte (Opinion: 1249937), strictly respecting the ethical principles contained in the Declaration of Helsinki. All the participants and their respective guardians were informed about the research objectives and the methods to be adopted in the study. Signed, informed consent (TALE and ICF) were obtained from the participants and their respective legal guardians in accordance with the Resolution 466/12 of the Ministry of Health (Brazil). This study complied with all the international requirements and standards of the STROBE checklist for observational studies of incidence and prevalence (i.e., checklist for the strengthening the reporting of observational studies in epidemiology) [28].

Research participants attended the lab on three occasions. The first visit was used to inform the individuals and their guardians about the research objectives and the methodological procedures adopted in the study. At the second visit, blood samples were taken for hormonal analysis and anthropometric measurements. On the third visit the children performed physical tests.

### 2.2. Anthropometry

All assessments were based on the International Society of Advancement of Kinanthropometry (ISAK) protocol, body mass was measured using a 0.10 kg digital scale (Filizola^®^, São Paulo, Brazil). Height was assessed using a 0.01 cm stadiometer (Sanny^®^, São Paulo, Brazil). Skinfolds were measured using a scientific adipometer (Harpenden^®^, John Bull Indicators Ltd., London, England). The perimeter was measured using an anthropometric tape (Sanny^®^, São Paulo, Brazil) and for the diameters a caliper (Sanny^®^, São Paulo, Brazil) was used [29]. 

### 2.3. Maturation

Skeletal maturity was determined using bone age (BA) in relation to chronological age according to the equation developed by Cabral et al. [30]. The mathematical model has been validated based on the gold standard for evaluation of bone age (X-ray of hand and wrist) and offers advantages of a high reliability level (r = 0.868; r² = 0.754) for being noninvasive, cost-effective, and nonexposure to radiation risks (X-ray). The mathematical model consists of the formula:

Bone age = −11.620 + 7.004 * (height) + 1.226 * (Dsexo) + 0.749 * (age) − 0.068 * (Tr) + 0.214 * (Cac) − 0.588 * (HD) + 0.388 * (FD).(1)

Height (cm); for the male Dsexo = 0, for the female Dsexo = 1, the chronological age in years, triceps skinfold (Tr), Corrected arm circumference (Cac), humerus diameter (HD), femoral diameter (FD).

The somatic maturation was verified by the predictive equations of Mirwald et al. [31], where the formulas measure and classify the peak of the speed of growth (PGS) in relation to the chronological age, in this way the PGS is determined through the following models’ equations.

PGS in male sex = −9.236 + [0.0002708 * (LL*TH)] + [−0.001663 * (A* LL)] + [0.007216 * (A*TH)] + [0.02292 * (W/E)*100](2)

PGS in female sex = −9.376 + [0.0001882 * (LL *TH)] + [0.0022 * (Ax LL)] + [0.005841 * (A*TH)] − [0.002658 * (A*W)] + [0.07693 * (W/E)*100](3)

LL = Leg length; TH = Trunk Height; A = Age; W = Weight; E = Estature.

Based on the final values of the equation results, in relation to chronological age, participants can be classified into three stages: (1) Pre-PGS (PGS < −1); (2) During PGS (PGS ≥ −1 or PGS ≤ + 1); and (3) Post-PGS (PGS > +1).

### 2.4. Upper Limb Power (RMPUL)

In this study, the upper limb power (RMPUL) was analyzed using the medicine ball test [32]. The participants were asked to sit with their back against a wall and knees extended. At the evaluator’s signal, a 2-kg medicine ball (Ax Sports^®^, Tangará, Brazil) was positioned at the height of the sternum was to be thrown horizontally using both hands. The aid of the trunk movement was not allowed. The test was performed consecutively for 3 times interspersed with a passive recovery period of 3 min. The best attempt measurement was considered for the analysis.

### 2.5. Lower Limb Power (RMPLL)

Lower limb power (RMPLL) was assessed by a vertical jump against movement using a jump mat (Cefise^®^, São Paulo, Brazil) connected to the Jump Test Pro 2.10 software (Cefise^®^, São Paulo, Brazil) [33]. To reduce errors during the execution of the protocol, participants were familiarized with the test. Starting from an orthostatic position, held for 3 s, with the knees flexed at approximately 90º and the hands fixed on the waist, the participants were instructed to perform the jump as high as possible. Three attempts were to be made, interspersed with 40 s of passive recovery, and the best attempt measurement was included for data analysis.

### 2.6. Upper Limb Speed (ULS)

For the assessment of upper limb speed (ULS), the subjects performed the tapping test, in which the participant uses the dominant hand in the shortest possible time [34]. The test is performed with the participant in front of a table of adjustable height to the hip level so that the nondominant hand is positioned in a central rectangle drawn on the table. The participants then touch the discs drawn on the sides of the table with their dominant hand. In this study, each participant performed 25 cycles between one side disc and another in the shortest possible time.

### 2.7. Body Speed with Change of Direction (BScD)

The body speed test with change of direction (BScD) was conducted according to the recommendations of Nimphius et al. [35]. Two vertical lines were drawn on the ground with a distance of 10 m between them and the central point was positioned in the middle of the 5-m distance marked on the ground with the drawing of a circle. This helped the participants run as fast as they could from the circle, touch the left line, change the direction, touch the right line, and return to the central circle, immediately starting the same procedure again 3 times in a row. This protocol was performed in 2 attempts, interspersed with a 3-min passive recovery time, and the best attempt measurement was considered for data analysis.

### 2.8. Analysis of Hormone Levels

Peripheral blood samples (10 mL) from the antecubital vein were obtained from the participants for analysis of hormone levels. The blood sample tubes were centrifuged with clot activator at 6000 rpm for 10 min to obtain 0.5 mL of serum. The serum samples were kept on ice at 20 °C and followed directly (the transport lasted 5 min) for the analysis of serum dosage of the hormones (testosterone and estrogen of the type estradiol). Subsequently, testosterone and estradiol measurements were carried out in nanomoles (nmol). The levels of said hormones also were measured using the direct chemiluminescence method, (i.e., reduction of light as a result of a chemical reaction in a blood sample) with the ADVIA Centaur^®^ XP – SIEMENS, Joinville, Brazil (i.e., Photomultiplier). The process transforms the light emitted by the chemiluminescence method into electrical impulses and thus the impulses are read in “count” of light per second (i.e., this unit is proportional to the unit of measurement of the hormone levels present in the sample). 

### 2.9. Statistics

To estimate the sample power and reduce the chances of type II error, a sample calculation was performed using the G. Power 3.01 software (University of Dusseldorf®, Dusseldorf, Germany). Considering that the previous conditioning promoted an effect size of 0.37, an analysis of variance of repeated measures, adopting α = 0.05 and β = 0.80, estimated the minimum number of 43 participants; in this sense, the sampling power was estimated at 0.80 [22]. Data normality was verified by Shapiro–Wilk and z-score tests for asymmetry and kurtosis (−1.96 to 1.96). Correlations were performed with nonparametric data by the Spearman test. For the partial correlations, the effect of the variables was controlled: Peak growth speed; bone age; estradiol; and testosterone. For comparisons, the data were transformed into parametric by the square root and subsequently the ANOVA One-Way Test was performed, followed by Tukey post hoc. The magnitude of the results of each correlation was determined by the scale proposed by Schober et al., [36]: Insignificant: r < 0.10; Weak: r = 0.10–0.39; Moderate: r = 0.40–0.69; Strong: r = 0.70–0.89; Very strong: r = 0.90–1.00. The data were transformed into parametric values by the square root, followed by linear regression analysis. Subsequently, regression analysis was performed. The homogeneity of the regression models was tested using the Breush–Pegan test and the assumptions of normality, variance, and independence of the data were not denied. Multilayer perceptron (MLP) nonlinear artificial neural networks were programmed using backpropagation algorithms. MLPs aimed to learn patterns of the relationships between neuromotor variables and hormonal markers and biological maturation variables, thus the networks were trained in multiple sessions to execute the algorithms to specify the relative probability of errors and hits for the possibility of prediction of neuromotor performance through the patterns of hormonal markers and biological maturation variables. All analyses were performed in the statistical software R (version 3.6.2, R Foundation for Statistical Computing®, Vienna, Austria), and was considered the significance level of *p* < 0.05.

## 3. Results

Table 1 reports the characterization of the sample. Describing in a descriptive way that the evaluated participants presented bone age similar to chronological age, a peak of growth speed with classification during peak of growth and estradiol levels more abundant than the levels of testosterone.

Table 2 presents the correlation matrix between hormones and maturation and strength performance. Estradiol is related to the maturation process of girls, as well as power performance. On the other hand, testosterone was not related to the peak growth rate, but was related to power capacity. In relation to the variables of upper limb velocity and body velocity with change of direction, there were no significant correlations. The analyses controlling the effect of the maturation variables on the relationship with the hormonal markers did not show statistical significance. In relation to somatic maturation, 17 subjects were classified into pre-peak growth velocity, 22 during-peak growth velocity and 5 post-peak growth velocity.

**Figure 1:**
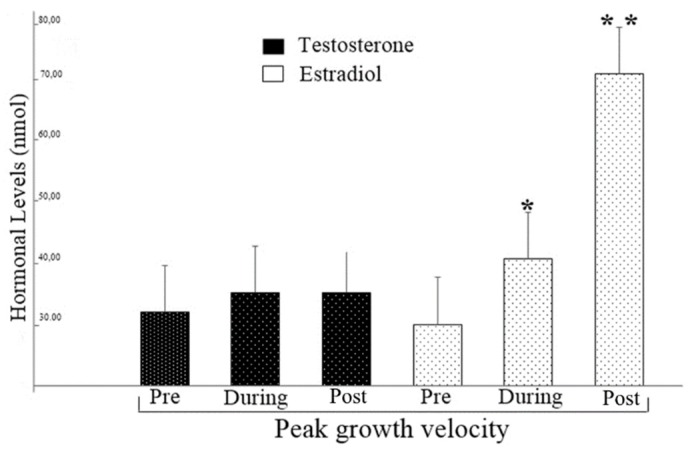
Comparison between groups regarding hormone concentrations. * = Superior to the group Pre peak of the speed of growth; ** = Superior to the Groups pre and during peak of the speed of growth.

Figure 1 shows the hormonal levels according to the maturation stage and it was evident that estradiol levels tend to increase significantly with advancing biological maturation (F _(41,0) =_ 8.13; *p* = 0.001), whereas for Testosterone concentrations did not show significant differences between maturational stages (F _(41,0)_ = 0.75; *p* = 0.4).

Table 3 presents the correlation matrix between maturation and strength performance. Peak speed growth is related to lower and upper limb power production capacity. The same was true for bone age. No relationship was found between maturation and the variables of upper limb velocity and body velocity with change of direction. When controlling the effect of hormonal markers on the relationship of neuromotor variables with those of maturation, it was found correlation for the power of upper and lower limbs remained statistically significant.

The regression models of maturational and neuromotor variables along with estradiol and testosterone levels are detailed in Table 4. The peak of the speed of growth, bone age, and neuromotor performance of lower limbs were found to be statistically significant.

The regression models of hormonal and neuromotor variables with skeletal and somatic maturation are shown in Table 5, so the analyses were statistically significant for estradiol levels, and for lower and upper limb potency. 

Table 6 reports that the perceptron artificial neural networks concluded with the results that skeletal maturation (bone age) has more than 50% probability of being able to predict the strength of the upper and lower limbs, and the somatic maturation (peak of the speed of growth) indicates a potential of more than 60% to anticipate the performance of upper and lower limbs, the speed of upper limbs, and the change of body direction at high speed. Yet, hormonal markers indicate a more than 95% chance of foreshadowing the strength of upper limbs.

## 4. Discussion

The aim of the present study was to verify the relationship between hormonal markers and biological maturation markers in the neuromotor abilities of female children. Similar to the objective of the present study, Lowe et al., [37] addressed that hormonal markers in the group of estrogens including estradiol interact with strength levels in women throughout life, and Aslam et al., [8] recently reported that there is a relationship between hormone levels during puberty and neuromotor skills in subjects of both sexes. In the same context to the objective of the present study, seeking to find relationships between maturation and neuromotor performance, Volver et al., [27], in a longitudinal study, analyzed 34 female subjects aged between 11 and 13 years old and, the authors concluded that sexual maturation influences neuromotor skills suggesting a possible interaction of factors related to puberty, such as hormone levels for example.

Thus, the results of the present study corroborate with the aforementioned studies and also with the statements of Sadiq et al., [10], where the authors highlighted that biological maturation is available for hormonal markers and that they can be used with different methods of the human body, cardiovascular, neurological and musculoskeletal system.

In this sense our data suggest that maturation is related to sex hormones, but in girls testosterone is not a major factor in peak growth velocity (Figure 1) and that both maturation and hormones are related to strength development in girls; however, they were not related to upper limb velocity and body velocity with change of direction (Table 2 and Table 3). The maturational process develops endocrine tissues to the point where their hormonal production capacity is high, near the growth spurt [4]. Moreover, the development of the hypothalamus-pituitary-gonads and hypothalamus–pituitary–adrenal axis act on the development of sexual characteristics (i.e., sexual maturation) [1] and changes in body composition [38]. However, as shown in Figure 1 of the results of the present study, this relationship between hormones and maturation should not be analyzed solely by chronological age, since there may be differences in hormonal responses in children of the same age due to distinct maturational stages [22].

Moreover, a relationship between hormone concentrations (Table 2) and biological maturation assessed by different methods (Table 3) and the level of muscle strength was demonstrated. In fact, maturation induces changes in neuromotor behavior in young people, mainly related to force [39]. In addition to hormonal changes, changes in muscle composition, energy metabolism, and neural develop during maturation and together develop the ability to produce strength [23].

Given this assumption, our findings indicate that with the advancing maturation, the levels of estradiol (female sex hormone) tend to increase (Figure 1). Our findings differ with the study by Pinto et al. [40], where the authors evaluated 89 children of both sexes aged between 10 and 13 years. The data from that study pointed out that for male and female participants, there were no statistical differences in relation to hormone levels with advancing sexual maturation stages. However, it was observed that in neuromotor skills there were no significant differences between the maturation stages of males, while in females, maturation stages III and IV (advanced) were superior to I and II (delayed) in relation to muscle power of the upper limbs.

It is noteworthy that in our results the neuromotor capacities showed correlations with the maturation evaluated by different protocols both for the power of upper limbs (bone age: r = 0.55; *p* < 0.0001; Peak growth velocity: r = 0.59; *p* < 0.0001) as for the power of lower limbs (bone age: r = 0.58; *p* < 0.0001; Peak growth velocity: r = 0.57; *p* < 0.0001). In this way, the power of upper limbs corroborated with the results found by Pinto et al. [22], where the authors found significant correlations of maturation with the muscular power of upper limbs but found no significant data for lower limbs potency. Júnior et al. [41] in their study also evaluated the power of lower limbs in a sample consisting of 46 school athletes of both sexes (aged 12 ± 3 years), and did not find results with significant values (*p* = 0.08), diverging from our findings. 

In regard to the correlation between bone age and estradiol, our data (bone age x estradiol: r = 0.58; *p* = 0.007) were positive corroborating with those of Pinto et al., [22] where a strong correlation of estradiol with bone age (r = 0.51; *p* = 0.001) was exposed. Therefore, it is noteworthy that in relation to testosterone our findings evidenced that the bone age also had a strong correlation (r = 0.51; *p* = 0.005), and this can be justified by the statements of Forrest [42] and Wirth et al., [43], the authors discuss that the testosterone has participation in bone development, influencing osteogenesis in particular during growth. 

In relation to the hormonal levels, the muscular power of the lower limbs (estradiol: r = 0.52; *p* = 0.007; testosterone: r = 0.42; *p* = 0.03) and higher (estradiol: r = 0.52; *p* = 0.006; testosterone: r = 0.42; *p* = 0.02) in our data were presented relations for estradiol levels; and for testosterone levels, in relation to estradiol the results found by the present study diverge from the results of Pinto et al., [22], where in relation to estradiol the correlation was not significant for the muscular power of limbs inferior (r = 0.15; *p* = 0.3) and it was significant for upper limb power (r = 0.37; *p* = 0.01).

The regression models of the study (Table 4 and Table 5) indicated that estradiol levels could assist in predicting peak growth speed, bone age, and lower limb neuromotor performance in the study participants. However, caution should be exercised in interpreting the values because regression determination coefficients revealed trivial magnitudes. In this study, testosterone levels did not indicate significant results in relation to the statistical regression models. However, analyses of artificial neural networks (Table 6) indicate that maturation can predict neuromotor performance by more than 50% and that hormonal markers have a potential of more than 95% for the probability of correct answers in relation to the prediction of upper limb performance.

In view of this approach, it is worth mentioning that hormonal levels influence the acquisition of lean mass and that lean mass is closely linked to the production of strength during the execution of neuromotor skills [4,23,27,44,45], and in this context young people with biological maturity accelerated body weight, greater concentration of lean mass, and a higher concentration of hormone levels [1,22,23,46]. This fact can also be confirmed in the results of the present study, corroborating the findings of Goswami et al. [26], where the authors showed that the muscular power of the lower and upper limbs is associated with maturation and that this association is significantly influenced by sex hormones, and that discriminating that with advancing maturation stages, the concentrations of hormone levels tends to increase.

In this sense, given that muscle strength is associated with the performance of various sports [44], autonomy from daily living activities, biomechanical functionality and that higher levels of strength are related to decreased risk of cardiovascular disease [47]. The findings of the present study are important to widen knowledge on how muscle strength is built until adulthood, explaining the importance of awareness of the maturation stage in young people. Considering that hormone levels can interact with the acquisition of lean mass and neuromotor performance, it is necessary to understand that individuals with similar chronological ages can have different levels of maturation and this can directly interfere with hormone levels (Figure 1).

Therefore, the future perspectives are that health and sport professionals have a particular perspective at the maturation process, using the findings discussed in this research in their professional practice. Thus, a possible practical applicability aimed at health professionals is the monitoring of maturation stages during interventions involving hormonal therapies (for young people who have growth deficit, or have a muscle strength profile below that expected that interferes with daily functionality). Sports professionals can use maturation assessment to monitor strength levels and associate them with the possible stage of neuromotor development that the participants find themselves in a non-invasive manner and with a low financial cost.

However, despite the relevance of the results, the present study has some limitations: Firstly, the fact that the study design is observational, which does not allow establishing a cause and effect relationship. Secondly, it was not possible to perform a longitudinal follow-up during the puberty period of the analyzed participants. Thirdly, the sample was made up only of female participants. Therefore, it is important to carry out new studies where there may be a longitudinal assessment and in both sexes. 

## 5. Conclusions

It was possible to concluded that estradiol hormone is related to maturational development in young girls and that both estradiol and testosterone showed positive relationships with bone age; The same was observed in relation to the performance of neuromotor capacities related to muscle power. While for the capacity of speed of the upper limb and speed of the body with change of direction, the hormonal markers did not present significant relation in the analyzed sample. The same results it was observed the relationship of biological maturation on neuromotor capacities. Thus, the hypothesis that the maturational stages and levels of hormonal markers in the organism can relate significantly to the performance of neuromotor abilities was confirmed.

## Figures and Tables

**Table 1:** Sample characterization.

Variables	Values
Sample (*N*)	44 (100%)
Weight (kg)	41.90 ± 8.34
Body mass index (m²)	19.04 ± 5.03
Heigth (cm)	1.49 ± 6.20
Cronological age	11.5 ± 1.5
Bone age	11.7 ± 2.3
Peak of speed growth	0.96 ± 0.3
Estradiol levels (nmol)	52.89 ± 4.56
Testosterone levels (nmol)	36.98 ± 4.27

**Table 2:** Correlation matrix between hormones and maturation and physical aspects.

Variables	Estradiol (nmol)		Testosterone (nmol)	
	r	*p*-Value	r	*p*-Value
Peak growth speed	0.51	0.004	0.22	0.14
Bone age	0.58	0.0007	0.51	0.005
RMPLL (W/kg)	0.52	0.006	0.42	0.03
RMPUL (cm)	0.51	0.007	0.42	0.02
ULS (s)	–0.007	0.9	–0.07	0.6
BScD (s)	–0.21	0.1	–0.01	0.9
**Peak of speed growth**				
RMPLL (W/kg)	0.18	0.2	0.16	0.2
RMPUL (cm)	0.16	0.2	0.17	0.2
ULS (s)	0.07	0.6	0.06	0.6
BScD (s)	0.14	0.3	0.05	0.7
**Bone age**				
RMPLL (W/kg)	0.15	0.3	0.10	0.4
RMPUL (cm)	0.21	0.1	0.13	0.4
ULS (s)	0.21	0.15	0.06	0.6
BScD (s)	0.13	0.3	0.08	0.6

r = Correlation coefficient; RMPLL = Relative muscle power of lower limbs; RMPUL = Relative muscle power of upper limbs; ULS (s) = Upper Limb Speed; BScD (s) = Body speed with change of direction.

**Table 3:** Correlations of peak growth velocity and bone age with physical variables.

Variables	Peak of Speed Growth		Bone Age	
	r	*p*-Value	r	*p*-Value
Estradiol (nmol)	0.51	0.004	0.22	0.14
Testosterone (nmol)	0.58	0.0007	0.51	0.005
RMPLL (W/kg)	0.57	<0.0001	0.58	<0.0001
RMPUL (cm)	0.59	<0.0001	0.55	<0.0001
ULS (s)	0.08	>0.5	−0.05	0.7
BScD (s)	−0.15	0.3	−0.14	0.3
Estradiol				
RMPLL (W/kg)	0.48	0.001	0.48	0.0009
RMPUL (cm)	0.51	0.0004	0.45	0.002
ULS (s)	0.12	0.4	0.14	04
BScD (s)	0.09	0.5	0.11	0.4
Testosterone				
RMPLL (W/kg)	0.53	0.0002	0.52	0.0002
RMPUL (cm)	0.55	0.0001	0.49	0.0008
ULS (s)	0.08	0.6	0.05	0.7
BScD (s)	0.19	0.2	0.21	0.1

r = Correlation coefficient; RMPLL = Relative muscle power of lower limbs; RMPUL = Relative muscle power of upper limbs; ULS (s) = Upper Limb Speed; BScD (s)= Body speed with change of direction.

**Table 4:** Linear regression of the study variables with hormonal markers.

Variables	Estradiol _(nmol)_				Testosterone _(nmol)_			
	r^2^	β	F _(42)_	*p*-Value	r^2^	β	F _(42)_	*p*-Value
Peak of the speed of growth	0.17	17.9	8.76	0.005	0.00	1.55	0.42	0.5
Bone age	0.30	19.5	18.1	0.0001	0.05	2.91	2.26	0.1
RMPLL (W/kg)	0.16	0.04	8.56	0.005	0.03	0.00	1.71	0.1
RMPUL (cm)	0.05	28.0	2.40	0.1	0.05	10.2	2.5	0.1
ULS (s)	0.00	−0.00	0.30	0.5	0.00	0.00	0.00	0.9
BScD (s)	0.05	−16.5	2.29	0.1	0.00	0.00	0.00	0.9

r² = regression determination coefficient; β = Angular regression coefficient in relation to the dependent variable; F = Value of the global significance of the regression; RMPLL = Relative muscle power of lower limbs; RMPUL = Relative muscle power of upper limbs; ULS (s) = Upper Limb Speed; BScD (s) = Body speed with change of direction.

**Table 5:** Linear regression of the study variables with maturation.

Variables	Bone Age				Peak of the Speed of Growth			
	r^2^	Β	F _(42)_	*p*-Value	r^2^	β	F _(42)_	*p*-Value
Estradiol (nmol)	0.30	0.01	18.1	0.0001	0.17	0.00	8.76	0.005
Testosterone (nmol)	0.05	0.01	2.26	0.1	0.00	0.00	0.42	0.5
RMPLL (W/kg)	0.38	0.12	26.2	<0.0001	0.26	0.08	14.8	0.0003
RMPUL (cm)	0.29	1.83	17.4	0.0001	0.29	1.50	17.2	0.0001
ULS (s)	0.02	−0.14	0.94	0.3	0.03	−0.14	1.31	0.2
BScD (s)	0.03	−0.39	1.66	0.2	0.02	−0.27	1.14	0.2

r² = regression determination coefficient; β = Angular regression coefficient in relation to the dependent variable; F = Value of the global significance of the regression; RMPLL = Relative muscle power of lower limbs; RMPUL = Relative muscle power of upper limbs; ULS (s) = Upper Limb Speed; BScD (s) = Body speed with change of direction.

**Table 6:** Analysis of artificial neural networks perceptron for the probabilities of predictions.

Variables	Bone Age			Peak of the Speed of Growth			Estradiol			Testosterone		
	U	%E	|P %	U	%E	|P %	U	%E	|P %	U	%E	|P %
RMPLL (W/kg)	3.54	35.2	64.8	2.88	27.6	72.4	6.68	94.4	5.60	6.18	98.4	1.60
RMPUL (cm)	2.54	42.6	57.4	1.12	24.0	76.0	0.48	4.31	95,6	0.94	4.57	95.4
ULS (s)	3.76	53.4	46.6	0.19	34.6	65.4	3.44	62.6	37.4	4.93	62.7	37.3
BScD (s)	3.43	61.2	38.8	−1.38	38.9	61.1	2.29	85.3	14.7	3.42	85.4	14.6

U = Mastery of the neural network activation function; % E = Percentage of total error of neural network learning; |Р **%** = Percentage of probability of prediction is correct; RMPLL = Relative muscle power of lower limbs; RMPUL = Relative muscle power of upper limbs; ULS (s) = Upper Limb Speed; BScD (s)= Body speed with change of direction.
